# Advancements in navigational bronchoscopy for peripheral pulmonary lesions: A review with special focus on virtual bronchoscopic navigation

**DOI:** 10.3389/fmed.2022.989184

**Published:** 2022-10-10

**Authors:** Mohan Giri, Haiyun Dai, Anju Puri, Jiaxin Liao, Shuliang Guo

**Affiliations:** ^1^Department of Respiratory and Critical Care Medicine, The First Affiliated Hospital of Chongqing Medical University, Chongqing, China; ^2^Department of Nursing, The First Affiliated Hospital of Chongqing Medical University, Chongqing, China

**Keywords:** bronchoscopy, peripheral pulmonary lesions, lung nodules, navigational bronchoscopy, virtual bronchoscopic navigation, interventional pulmonology, radial probe endobronchial ultrasound, robotic bronchoscopy

## Abstract

Lung cancer is often diagnosed at an advanced stage and is associated with significant morbidity and mortality. Low-dose computed tomography for lung cancer screening has increased the incidence of peripheral pulmonary lesions. Surveillance and early detection of these lesions at risk of developing cancer are critical for improving patient survival. Because these lesions are usually distal to the lobar and segmental bronchi, they are not directly visible with standard flexible bronchoscopes resulting in low diagnostic yield for small lesions <2 cm. The past 30 years have seen several paradigm shifts in diagnostic bronchoscopy. Recent technological advances in navigation bronchoscopy combined with other modalities have enabled sampling lesions beyond central airways. However, smaller peripheral lesions remain challenging for bronchoscopic biopsy. This review provides an overview of recent advances in interventional bronchoscopy in the screening, diagnosis, and treatment of peripheral pulmonary lesions, with a particular focus on virtual bronchoscopic navigation.

## Introduction

The global burden of cancer incidence and mortality is increasing at an alarming rate. Lung cancer is the leading cause of cancer-related death, accounting for ~1.8 million deaths in 2020 (1). Therefore, early screening, diagnosis, and treatment play a vital role in improving the survival rate of lung cancer patients. Low-dose computed tomography (LDCT) has been increasingly accepted for screening of lung cancer which has resulted in a significant increase in the detection rate of peripheral pulmonary lesions (PPLs) in recent years (2, 3). Although there is no precise definition, peripheral pulmonary lesion, also known as peripheral lung lesion, is a lung nodule measuring ≤ 3 cm in diameter and located in the lung periphery (4). PPLs pose diagnostic challenges for oncology, radiology, thoracic surgery, and pulmonary medicine specialists, especially for nodules that are often smaller. The British Thoracic Society (BTS) (4) and Fleischner Society (5) guidelines that incorporate the opinions of a multidisciplinary team of thoracic radiologists, pulmonologists, surgeons, pathologists, and other specialists all over the world are two of the most widely used guidelines for the investigation and management of pulmonary nodules. Pulmonary lesions with CT characteristics suggestive of malignancy require CT surveillance, CT-guided biopsy, image-guided bronchoscopic biopsy, and surgical resection to provide a definitive diagnosis. The era of bronchoscopy began with Gustav Killian in 1876, and the development of the flexible fiberoptic bronchoscope by Shigeto Ikeda in the late 1960 has revolutionized the field of bronchoscopy (6). For the first time, Leira et al. employed a mechanically ventilated pig model to show the impact of the wedging technique in navigated bronchoscopy. Wedging the bronchoscope in segmental airways induced significant displacement (0.9–17.8 mm) of peripheral lesions in a pig model (7).

The last decade has had exponential growth in the arena of diagnostic bronchoscopy. Percutaneous needle biopsy and CT-guided transthoracic needle aspiration (CT-TTNA) are recommended modalities for tissue diagnosis of pulmonary lesions, but both have limitations. Following the CT-guided transthoracic needle aspiration, pneumothorax is the most common complication (8, 9). The diagnostic rate of transbronchial biopsy for small lesions in diameter of ≤ 20 mm is not satisfactory. In recent years several guided bronchoscopy technologies such as radial EBUS (r-EBUS), ultrathin bronchoscopy, virtual bronchoscopic navigation (VBN), electromagnetic navigation bronchoscopy (ENB), transparenchymal nodule access (TPNA), and robotic bronchoscopy have been developed to assist in the biopsy of peripheral pulmonary lesions (PPLs). This review article aims to summarize the most recent data available on these guided bronchoscopy technologies, highlight the advantages and disadvantages of these technologies, and look ahead to potential future technologies.

## Search strategy

A literature review was conducted using PubMed, Embase, Scopus, and web of science (WOS) databases to identify relevant English-language articles published from inception through March 1, 2022. The search terms included “navigation bronchoscopy”, “virtual bronchoscopic navigation”, “robotic bronchoscopy”, “transbronchial biopsy” and “ultrathin bronchoscope” in combination with “peripheral pulmonary lesions”, “peripheral pulmonary nodule”, and “lung nodule”. The search yielded 1,352 articles. The titles and abstracts were reviewed independently by the authors. We searched references of articles to identify additional relevant articles. Additionally, we searched the ClinicalTrials.gov trial registry to identify active clinical trials related to our search terms.

## Virtual bronchoscopic navigation (VBN)

**Figure 1:**
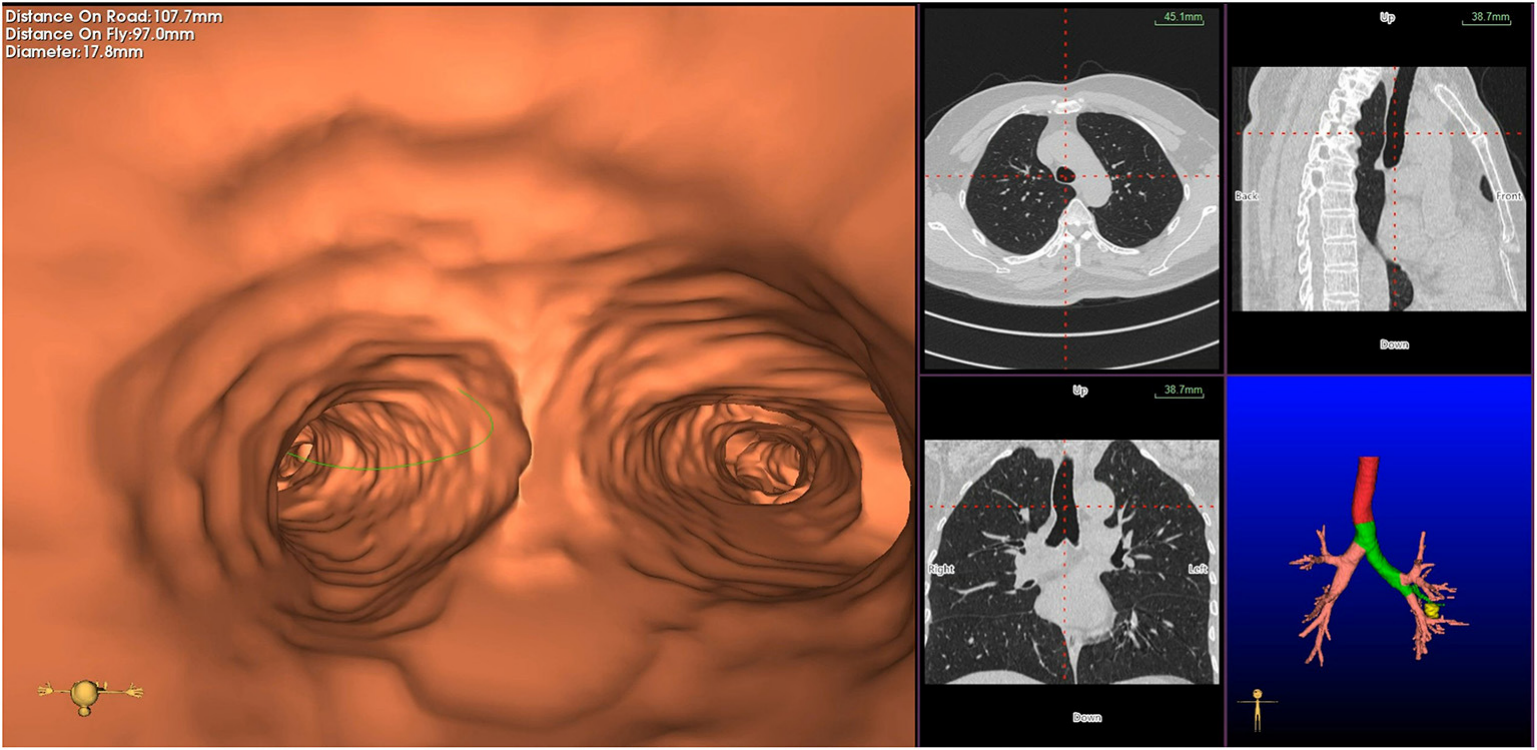
Virtual bronchial path (fly through) to the target peripheral pulmonary lesion (green line represents bronchial path).

**Figure 2:**
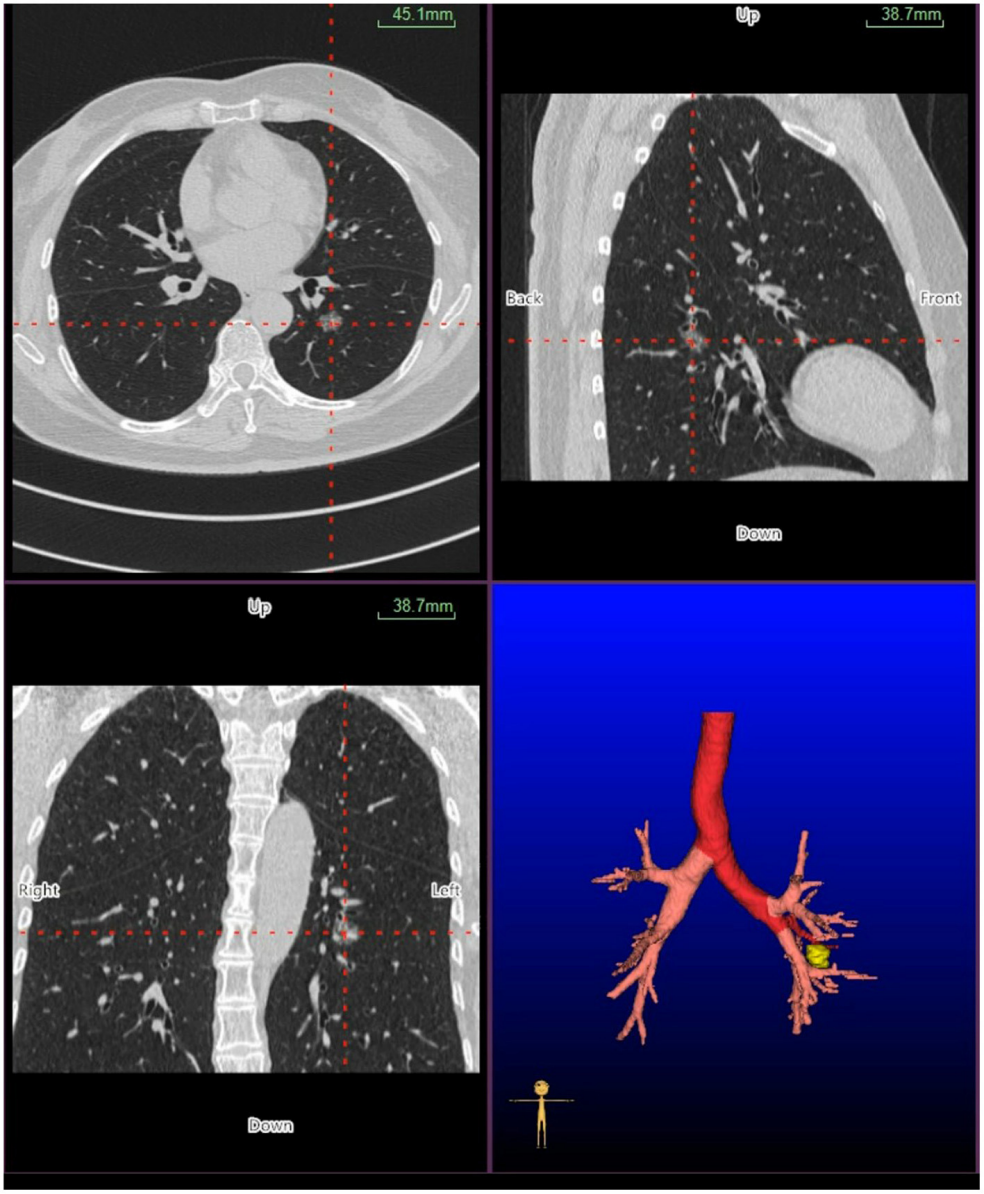
Planning phase- multi-planar views of the target peripheral pulmonary lesion (axial, sagittal, coronal, and 3D map).

**Figure 3:**
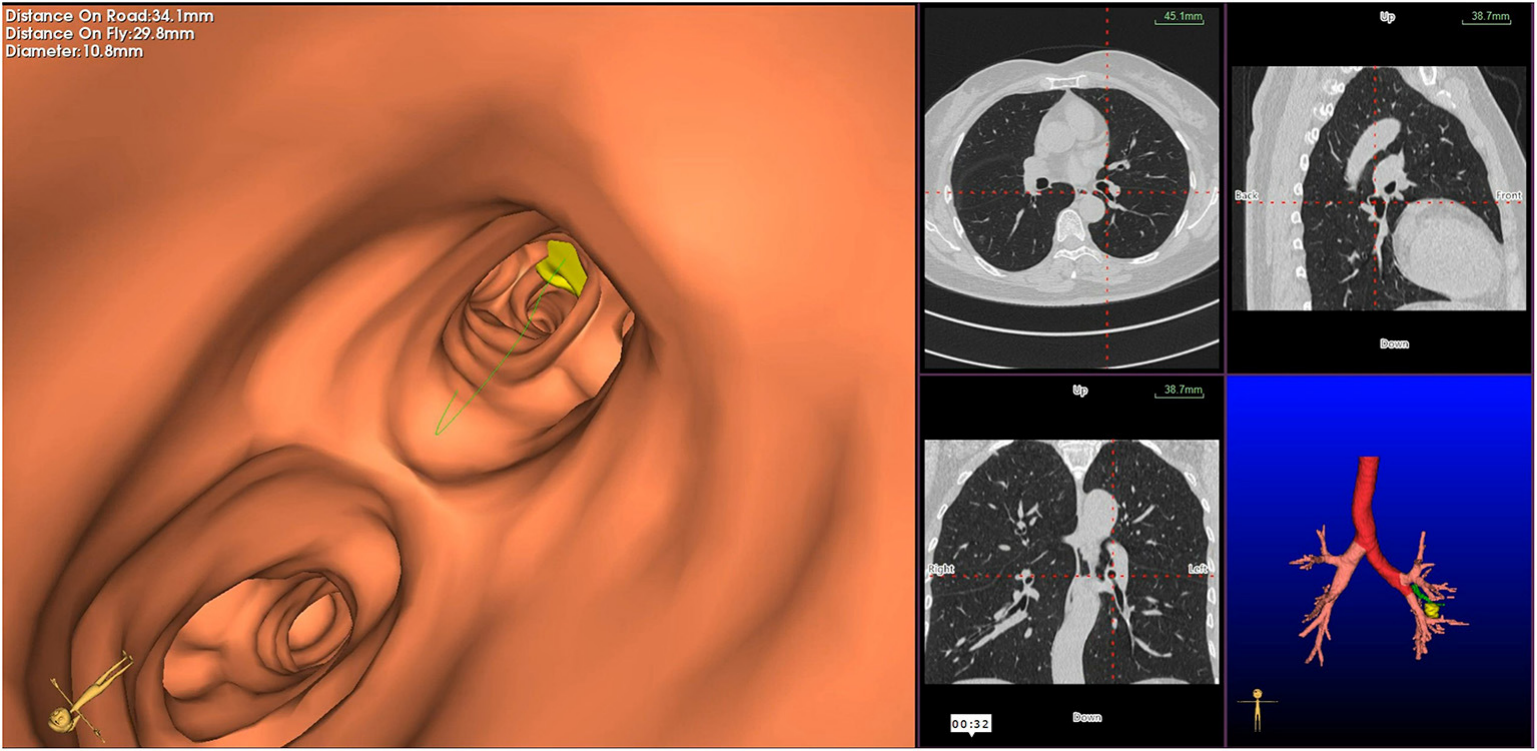
Virtual bronchoscopic image depicting the bronchial route to the target lesion—the green line represents the bronchial route to the lesion, and the yellow circle represents the target peripheral pulmonary lesion.

Virtual bronchoscopic navigation is a navigational technique that utilizes data of helical CT to construct three-dimensional virtual images of the bronchial route to guide the bronchoscope to the target lesion (Figure 1). Bf-NAVI^®^ (Cybernet System Inc., Tokyo, Japan) was the first VBN system that was introduced in Japan in 2008, LungPoint^®^ (Broncus Medical Inc., Mountain View, CA, USA) was launched in 2009 in the USA and is now widely used in the USA and European countries (10). DirectPath^®^ (Cybernet System Inc.), which has replaced Bf-NAVI, is used in China by most centers. There are mainly 3 phases of virtual bronchoscopic navigation: planning, guidance, and biopsy. **(i) Planning phase:** In the planning phase, data from multi-detector chest CT of patients are imported to the computer *via* VBN software (Figure 2), which automatically creates the virtual bronchoscopic pathway to the target lesion (Figure 3). To obtain high-quality virtual bronchoscopic (VB) images, continuous volume CT data is required. Planning is a pre-bronchoscopy process that is usually done on the same day or days before the intended biopsy. (**ii) Guidance phase:** The virtual images acquired during the planning phase are used to advance the bronchoscope manually to the target lesion, and the position of the bronchoscope tip can be displayed on the CT images corresponding to the bronchial tree. **(iii) Biopsy phase**: The choice of procedure to biopsy lesion depends on patient selection and target disease. PPLs can be sampled with flexible bronchoscopy, ultrathin bronchoscopy, and radial endobronchial ultrasound probes (RP-EBUS).

## VBN combined with other modalities to diagnose PPLs

VBN alone cannot guide a biopsy instrument to the target lesion, so VBN is generally used in combination with other bronchoscopic technologies. Here we summarize the results of studies that reported the combined use of VBN with CT, X-ray fluoroscopy, and endobronchial ultrasonography (EBUS) for diagnosing PPLs.

### VBN combined with R-EBUS/EBUS GS

The combined use of VBN and EBUS-GS exploration technology for diagnosing PPLs has widely been used across different bronchoscopic centers worldwide. A recent study by Liu et al. (11), which included 202 patients, found that using VBN and endobronchial ultrasonography using a guide sheath (EBUS-GS) shortened the time to locate the lesion. However, the total diagnostic rate and total examination time were similar in both the observation and control groups. VBN-assisted EBUS-GS without fluoroscopy guidance can be performed by the operator without the expertise and the overall diagnostic yield and malignancy diagnostic yield of 74.3 and 83.7% demonstrated that performance of VBN without fluoroscopy might be similar to that with fluoroscopy guidance in diagnosis of PPLs (12). In a prospective phase II study that evaluated the usefulness of combining EBUS, VBN, and ROSE to diagnose small PPLs, Maekura et al. could not demonstrate the usefulness of this combined method for diagnosing small PPLs (13). In conflict with previously reported studies (14, 15), Bae et al. (16) revealed that the diagnostic yield of VBN assisted endobronchial ultrasound guide sheath transbronchial lung biopsy (EBUS GS TBLB) for PLL did not improve in comparison with non-VBN assisted EBUS GS TBLB. In 105 patients with PPLs, Xu et al. (17) investigated the clinical value of virtual bronchoscopy navigation combined with EBUS-transbronchial lung biopsy (TBLB) in the diagnosis of PPLs without X-ray guidance, and they found that there was no significant difference between the VBN + EBUS group and the EBUS group (76.0 vs. 65.5%, *P* = 0.287) for the overall diagnostic rate, however, the VBN shortened the total examination time. Another study conducted by Matsumoto et al. (15) found that the ziostation group (VBN + EBUS) had a significantly higher diagnostic yield than the non-VBN group (77.7 vs. 64.6%, *P* = 0.030).

### VBN combined with computed tomography (CT)

Computed tomography (CT) can detect the small peripheral pulmonary lesions (PPL) that are hard to visualize with X-ray fluoroscopy. The prospective study (18) that examined the value of using a VBN system to assist CT-guided transbronchial biopsy (CT-TBB) in the diagnosis of PPL showed that the diagnostic yield of small PPL was 84.0% in the group with VBN plus CT-TBB vs. 58.0% for the group without VBN, which implies that VBN improves diagnostic yield for PPL with <20 mm in diameter but the total examination time did not differ between the two groups. Another study (19) which evaluated the safety and efficacy of CT-guided transbronchial biopsy (TBB) using an ultrathin bronchoscope with navigation by virtual bronchoscopy showed that the diagnostic sensitivity of this procedure was high for small peripheral pulmonary lesions as well as the total examination time was significantly shorter in the navigation group.

### VBN combined with ultrathin bronchoscopy

A Comparative study (20) that examined the diagnostic yield of VBN-guided and unguided ultrathin bronchoscopy (UTB) for PPLs revealed that the overall diagnostic yield did not differ between the VBN-guided and unguided arms. Furthermore, Biswas et al. (21) demonstrated that combining the virtual bronchoscopic approach to a peripheral lung lesion using a UTB yielded a diagnostic rate of 67.3% (35/52) without any procedural complications. Thus virtual bronchoscopy-guided ultrathin bronchoscopy is safe for evaluating peripheral pulmonary nodules. VBN is useful to guide the ultrathin bronchoscope to the target lesions with a high diagnostic yield, and Ali et al. (22) also found that the VBN and cone-beam computed tomography (CBCT) guided transbronchial biopsy had a high diagnostic yield for pulmonary nodules.

### VBN combined with X-ray and non-X-ray fluoroscopy

Diagnostic rates of 54.5% for lesions ≤ 20 mm and 94.7% for 21–30 mm lesions in the study by Tachihara et al. (23) showed that virtual bronchoscopic navigation under X-ray fluoroscopy was useful for the diagnosis of small PPLs. A prospective study (24) that evaluated the usefulness of combined techniques with VBN and EBUS-GS without x-ray fluoroscopy for small PPLs revealed that the overall diagnostic yield was 83.3% in the X-ray group and 69.2% in the non-X-ray group, however, this was a single-center study with small sample size.

## Randomized controlled trials on VBN for the diagnosis of PPLs

Our literature search yielded six randomized controlled trials (Table 1) that compared virtual and non-virtual bronchoscopic navigation for diagnosing peripheral pulmonary lesions. A prospective multicenter study (25) evaluated the value of VBN-assisted bronchoscopy and showed that there was no significant difference in the overall diagnostic yield between the VBNA group and the NVBNA group. However, in subgroup analysis, the VBNA group had a higher diagnostic yield than the NVBNA group for lesions in the right upper lobe, lesions that were not visible on P-A radiographs, and lesions in the peripheral third. Furthermore, complication rates were similar in both groups. Another randomized clinical trial (26) conducted at 5 Japanese medical centers that assigned patients to the VBNA or X-ray fluoroscopy-assisted (XRFA) groups showed no significant difference in the diagnostic yield between the groups for the diagnosis of peripheral pulmonary lesions. Moreover, there was no significant difference between the VBNA and (XRFA) groups in terms of total examination time. Bo et al. (27) conducted a randomized controlled trial involving 1,010 subjects with solitary pulmonary nodules using multiple bronchoscopic guided technologies. They found that guided bronchoscopy (combined EBUS-GS with VBN) had superior diagnostic yield in the context of peripheral lesions. Furthermore, PPLs with diameters >20 mm had a significantly higher diagnostic yield than those with diameters <20 mm.

**Table 1:** Randomized controlled trials on VBN for the diagnosis of peripheral pulmonary lesions.

**Study (year)**	**Study design**	**Country**	**Overall diagnostic yield**	**Brand of VBN**	**Bronchoscope used**	**Other auxiliaries used**	**Biopsy method**
Asano et al. (25)	Randomized controlled trial	Japan	112/167 (67.1%)	Bf-NAVI	XP260F, XP40	X-ray	Forceps, brush, lavage
Asano et al. (26)	Randomized controlled trial	Japan	50/65 (76.92%)	Bf-NAVI	P260F	EBUS-GS, X-ray	Forceps, brush, lavage
Bo et al. (27)	Randomized controlled trial	China	248/334 (74.25%)	DirectPath	Not available	EBUS-GS	Forceps
Chen et al. (28)	Randomized controlled trial	China	67/93 (72.04%)	DirectPath	BF-1 T260/ BF-F260	EBUS	Brush
Ishida et al. (14)	Randomized controlled trial	Japan	80/99 (80.80%)	Bf-NAVI	BF-P260F	EBUS-GS	Forceps, brush
Xu et al. (29)	Randomized controlled trial	China	46/55 (83.63%)	DirectPath	Olympus BF-P260F	EBUS	Forceps

VBN, virtual bronchoscopic navigation; EBUS-GS, endobronchial ultrasonography using a guide sheath.

The study that evaluated the diagnostic utility of EBUS and virtual bronchoscopic navigation combined with endobronchial ultrasound (VBN + EBUS) for the solitary pulmonary nodules revealed no significant difference in the diagnostic yield between the VBN + EBUS group and the EBUS group (83.6 vs. 66.7%, *P* = 0.419) (29). They also showed a higher diagnostic yield in the VBN + EBUS group than in the EBUS group for lesions with a diameter <20 mm. Another prospective RCT (14) conducted in three Japanese medical centers randomly assigned patients with peripheral pulmonary lesions to VBN-assisted (VBNA) or non-VBN-assisted (NVBNA) groups found that VBN-assisted EBUS had significantly superior diagnostic yield for small pulmonary peripheral lesions than the NVBNA group. Additionally, VBN shortened the total examination time without any severe or moderate adverse events associated with virtual bronchoscopic navigation. Chen et al. (28) evaluated the diagnostic value of EBUS-GS-TBLB with the VBN group and EBUS-GS-TBLB without the VBN group in 184 patients with peripheral pulmonary lesions. They found that overall diagnostic yield did not differ between the VBN-guided and unguided groups. However, VBN shortened the total examination time. VBN has also been used to guide thin and ultrathin bronchoscopes to the target peripheral lesions. Oki et al. (30) used VBN in conjugation with EBUS, fluoroscopy in ultrathin bronchoscope (UTB group), and thin bronchoscope with a guide sheath (TB-GS group) to compare the diagnostic yield of transbronchial biopsy. They concluded that the diagnostic yield of the UTB method was higher than that of the TB-GS method when both groups were guided with VBN and other auxiliaries. A recent RCT (31) compared rEBUS, virtual bronchoscopic navigation, and fluoroscopy-guided ultra-thin bronchoscope (UTB group) and thin bronchoscope (thin bronchoscope group) for the diagnosis of peripheral pulmonary lesions also demonstrated that the VBN-guided multimodal bronchoscopy with ultra-thin bronchoscope was superior to the thin bronchoscope in the diagnosis of small PPLs.

## Advantages and disadvantages of VBN

### Advantage of VBN

The advantages of VBN are as follows:

(I) VBN has a low cost compared with other novel bronchoscopic technologies such as electromagnetic navigation bronchoscopy and robotic bronchoscopy.
(II) VBN can guide the bronchoscope to the target lesion by creating a bronchial route in a short duration and reducing the radiation exposure to both the operator and patient.
(III) VBN can shorten the time needed for guidance as other auxiliary imaging techniques, such as fluoroscopy are not required.
(IV) VBN has technical simplicity; no specific training is required for the operator.
(V) VBN has lower complication rates than conventional transthoracic needle aspiration (TTNA).
(VI) Virtual image preparation may have educational benefits for inexperienced pulmonologists without prior experience with EBUS-GS or other imaging modalities.
(VII) VBN is a painless and non-invasive procedure.
(VIII) VBN has a high diagnostic yield for small PPLs ≤ 20 mm.
(IX) VBN can shorten total examination time, which is beneficial for patients undergoing procedures under local anesthesia.

### Disadvantages of VBN

The disadvantages of VBN are as follows:

(I) VBN images are formed automatically by VBN software, and high-quality CT images are important for the bronchial route to the target lesion. If the quality of CT images is poor due to respiratory artifacts, we cannot expect accurate VB images. Additional CT examination may be required in such a case, which increases the patient's radiation exposure and examination costs to a certain extent.
(II) The arrival of a bronchoscope to the target lesion cannot be confirmed by the VBN system alone.
(III) VBN alone cannot replace X-ray fluoroscopy because it reduces the accuracy and efficiency of sample collection from lesions.
(IV) Some studies reported that VBN could increase diagnostic yield for PPLs; however, VBN cannot improve the diagnostic sensitivity.
(V) In some cases, VBN guidance is limited by insufficient segmentation of the peripheral airways.
(VI) The VBN images used to guide a bronchoscope to the target lesion are taken before the bronchoscopy and can only reflect the bronchoscope's real-time situation to a certain extent. EBUS may thus be required to confirm real-time arrival at the target lesion.

## Recent advancements in navigational bronchoscopy for PPLs

Recent advances in interventional bronchoscopy have been successfully used to diagnose peripheral pulmonary lesions with superior diagnostic yield. Each modality has evolved into a pragmatic tool for clinicians to sample peripheral lung lesions.

### Electromagnetic navigation bronchoscopy (ENB)

Alike VBN, electromagnetic navigation bronchoscopy utilizes thin-section CT to create images of the virtual tracheobronchial tree. The electromagnetic field generated by the board placed around the patient's chest help as a sensor in the electromagnetic field and remunerates for respiratory movements. Two types of ENB systems are commercially available: the SuperDimension system (Medtronic, Minneapolis, MN, USA) and the SPIN Drive System (Veran Medical Technologies, St. Louis, MO, USA). The NAVIGATE study (32) that evaluated ENB using the superDimension navigation system among 1,157 patients undergoing ENB-guided biopsy showed that navigation was successful in 94.4% of patients, and malignancy was diagnosed in 44.3%. Furthermore, ENB-aided diagnosis was achieved in 66–75% of patients with lower complication rates even in the challenging lesion. A recent meta-analysis (33) that compared the sensitivity and safety of electromagnetic navigation bronchoscopy for lung cancer diagnosis reported that ENB had pooled sensitivity of 77% and a specificity of 100% for malignancy. McGuire et al. (34) also showed similar sensitivity for ENB (70.7%) in a meta-analysis that compared diagnostic accuracy and sensitivity for R-EBUS and ENB in sampling PPLs. AQuIRE registry (35), which compared electromagnetic navigation (EMN) with standard bronchoscopy in patients with peripheral lung lesions, found that the diagnostic yield of EMN alone was lower than in the previous study (36). A prospective randomized controlled trial conducted by Eberhardt et al. (37) demonstrated that combining EBUS and ENB improves the diagnostic yield of flexible bronchoscopy in PPLs when compared to either method alone [88% for combined approach vs. EBUS (69%) or ENB alone (59%)]. In terms of safety, there were no instances of bleeding that required interventions such as cold saline instillation or endobronchial blocker insertion. Ozgul et al. (38) investigated the diagnostic yield and safety of ENB with or without r-EBUS for peripheral lung lesions and demonstrated that the diagnostic yield in ENB plus r-EBUS was 73%, while it was 71.4% in the ENB group. They also concluded that ENB with or without r-EBUS is a safe and efficient method for sampling peripheral pulmonary lesions. The potential reasons for the discrepancy in diagnostic yield of EMN across various studies may be due to patient selection and the expertise level of the operator carrying out the procedure.

### Bronchoscopic transparenchymal nodule access (BTPNA)/transbronchial access tool (TBAT)

The bronchoscopic transparenchymal nodule access (BTPNA) is a novel technique to access peripheral pulmonary lesions that do not have a bronchus leading to them. BTPNA is still investigational, and trials that evaluate the diagnostic yield of the BTPNA are still in their infancy. Herth et al. (39) conducted the first human trial of BTPNA by creating a tunnel pathway in patients to evaluate the safety and feasibility of a novel approach for diagnosing solitary pulmonary nodules. They found that the BTPNA approach improved the accessibility of peripheral nodules in 10 out of 12 patients without any significant complications. Harzheim et al. (40) performed the BTPNA procedure successfully in 5 out of 6 patients in another prospective, single-arm interventional study. Even though there were no immediate complications during the procedure, pneumothorax developed in two patients two hours after the procedure. There is currently one prospective, multicenter, real-world study (NCT04597346) evaluating the safety and efficacy of bronchoscopic transparenchymal nodule access (BTPNA) for the diagnosis of peripheral lung lesions (41). This study will enroll 200 participants older than 18 years with peripheral pulmonary lesions that are difficult to reach or have no bronchus sign. The primary objective of this study is to analyze the overall diagnostic yield. The secondary outcome measures are the success rate of navigation, the success rate of biopsy, the operation time of the bronchoscope, the total time of arrival of the lesion, the intraoperative fluoroscopy time, factors affecting the diagnosis rate, factors affecting the biopsy success rate, and factors affecting the navigation success rate. The results of this study are anticipated in early 2023.

The transbronchial access tool (TBAT) is another technology to access peripheral pulmonary lesions in the absence of an air bronchogram. Bowling et al. (42) used ENB-guided biopsies and the TBAT to evaluate pulmonary lesions without air bronchogram on thoracic imaging among 14 patients and achieved access in 75% (9 of 12) of the targeted lesions, with a diagnostic yield of 66%. TBAT has also been found to be a valuable and safe diagnostic technique for diagnosing PPLs when used in conjunction with other modalities in other studies (43, 44). Although these new techniques appear to be promising for sampling lesions without a bronchus sign, more studies with a larger sample size are needed to evaluate the feasibility and safety of the BTPNA/TBAT procedure.

### Cone-beam computed tomography (CBCT)

Cone-beam computed tomography (CBCT) is a new emerging technology that provides near real-time high-resolution 2D fluoroscopy and volumetric CT images. Although this modality has been utilized in different fields of interventional radiology, such as intravascular and hepatobiliary interventions, there is limited existing data on this novel technique for diagnosing peripheral pulmonary lesions. A recent retrospective study (45) that compared the usefulness of the CBCT-guided TBB and CT-guided TBB using a propensity score-matched analysis revealed that the CBCT-guided group (72.9%) had a superior overall diagnostic yield than the CT-guided group (47.9%) for peripheral pulmonary lesions. Furthermore, the total examination time in the CBCT-guided group was significantly decreased than in the CT-guided group (*P* = 0.001). Ali et al. (22) combined CBCT with ultrathin bronchoscope and VBN to diagnose small pulmonary lesions and found that this combined guided CBCT has a high diagnostic yield for pulmonary nodules (90.0%). Furthermore, CBCT, combined with other bronchoscopic modalities, provides a higher diagnostic yield for peripheral lung nodules (46, 47). Moreover, a study that examined the overall diagnostic yield for PPLs using CBCT- augmented fluoroscopy combined with radial EBUS demonstrated superior diagnostic yield (75.5 vs. 52.8%) and reduced procedural time than that with EBUS alone (48). A study by Kheir et al. (49) compared the diagnostic yield of electromagnetic navigation bronchoscopy (ENB) plus CBCT as compared with ENB alone revealed that the diagnostic yield of ENB-CBCT and ENB was 74.2% and ENB 51.6%, respectively, for PPLs. In addition, ENB plus CBCT shortened procedural time compared with the ENB group. However, this was a retrospective study with a small sample size. These findings will need to be validated in future randomized clinical trials.

### Robotic bronchoscopy

To overcome the limitations of conventional and guided bronchoscopy for sampling PPLs, robotic-assisted bronchoscope systems have been developed in the hopes of targeting lesions that are beyond the reach of bronchoscopists. MonarchTM platform by Auris Health was the first robotic bronchoscopy that received clearance from the Food and Drug Administration (FDA) in 2018 for diagnostic and therapeutic bronchoscopic procedures. Another robotic bronchoscopy platform called the Ion system (Intuitive Surgical Inc. Sunnyvale, California, USA) got approval from the FDA for commercial use in February 2019. Fielding et al. (50) used the first-in-human robotic-assisted bronchoscope (Intuitive Surgical, Sunnyvale) system with a remotely controlled catheter to access small peripheral lesions. They demonstrated that the robotic-assisted bronchoscope system successfully obtained samples in 96.6% of cases, without any device-related complications. Furthermore, the overall diagnostic yield and yield for malignancy were 79.3 and 88%, respectively. The robotic bronchoscopic system's ability to reach and access artificial tumor targets in cadaveric lungs was evaluated in the ACCESS study (51). It was highly successful at biopsying peripheral lesions 10–30 mm in size. A recent prospective, multicenter study (52) on robotic bronchoscopy in patients with PPL has shown that the R-EBUS imaging probe introduced with the help of robotic scope localized target lesion successfully in 96% of patients with limited adverse events. In this study, 54 patients were enrolled, and the diagnosis was achieved in 74.1% of patients, while Malignancy accounted for 82.5% of those who received a diagnosis.

Additionally, pneumothorax was reported in two cases (3.7%) only. The first prospective randomized trial (53) of robotic bronchoscopy vs. EMN vs. rEBUS with an ultrathin bronchoscope in a human cadaveric model revealed successful localization and needle puncture of the single peripheral pulmonary nodule were significantly higher in the robotic bronchoscopy group than in other existing technologies. Benn et al. (54) combined robotic-assisted navigation bronchoscopy with cone beam CT to biopsy lung nodules in 52 patients with lung nodules and successfully reached all nodules. This combined modality achieved the definitive diagnosis in 83% (49/59) of biopsied nodules. There are three ongoing clinical trials (ClinicalTrials.gov) on robotic bronchoscopy for diagnosing peripheral lung nodules (NCT04182815, NCT04740047, and NCT04441749). These trials are expected to complete only after mid-2023. It is anticipated that robotic bronchoscopy will become a vital tool for diagnosing PPLs in the near future.

## Conclusion

Recently, significant advancements have been made in the field of bronchoscopy to diagnose peripheral lung lesions. Table 2 summarizes the advantages and disadvantages of recent bronchoscopy developments for diagnosing PPLs. VBN is an important tool that can be used alone or in conjugation with other bronchoscopic technologies to guide the bronchoscope to the target lesions. It is safe, painless, and less expensive, and the operator does not require expertise. It has a higher diagnostic yield for small PPLs ≤ 20 mm and can shorten the total examination time. The revolutionary developments in bronchoscopic modalities, including VBN, ENB, BTPNA/TBAT, and r-EBUS, cone-beam computed tomography, and robotic bronchoscopy, have improved diagnostic yield and successful localization of PPLs. However, these new techniques require further studies to establish their utility in clinical practice. Future multi-center, randomized, and prospective trial studies are warranted to provide more robust evidence to support the clinical application of navigation bronchoscopy for diagnosing PPLs.

**Table 2:** Summary of the advantages and disadvantages of bronchoscopic technologies for diagnosing PPLs.

**Bronchoscopic technologies**	**Advantages**	**Disadvantages**
Conventional transbronchial needle aspiration	1) Low-cost, safe, and less invasive.	1) Operator's experience is needed
		2) Inadequate sensitivity
		3) Systemic training is required because C-TBNA is done without real-time ultrasound guidance.
		4) A high cumulative radiation dose for both the patient and the operator
		5) Limited range of motion
CT-guided transthoracic needle aspiration	1) High diagnostic yields, even for small nodules.	1) Complications such as pneumothorax
	2) Excellent diagnostic yields for small lesions.	2) Not suitable lung lesions in the lower chest due to diaphragmatic movement.
Thin/ultrathin bronchoscopes	1) Easy access to peripheral pulmonary lesions	1) It Cannot obtain a larger specimen size
	2) Promising diagnostic yield	
Radial probe endobronchial ultrasound	Outperforms conventional bronchoscopy in terms of diagnostic rate and complications.	1) Higher costs, as well as timing and ultrasound-specific skill, is a must.
		2) Only available in higher centers and hospitals
Virtual navigation bronchoscopy	1) Compared to ENB, VBN is less expensive, and VBN is a simple technique, no special training is required to perform it.	1) VBN cannot be a substitute for X-ray fluoroscopy.
	3) Additional sensor or a specific biopsy instrument is not required.	2) VBN cannot improve diagnostic sensitivity.
	4) It has low complication rates	3) Inadequate segmentation of the peripheral airways limits VBN guidance.
	5) For PPLs ≤ 2 cm vbn has superior diagnostic rate.	
Electromagnetic navigation bronchoscopy	1) The real-time navigation of ENB helps to increase diagnostic yield	1) Each patient requires costly disposable instruments.
		2) ENB has a higher specificity but is invasive and may cause pain.
		3) Expertise training is required.
Robotic bronchoscopy	1) It can penetrate significantly deeper into the bronchial tree, especially in difficult airways.	1) Higher costs
	2) Can target lesions with more accuracy with fewer complications than conventional bronchoscopy techniques.	2) Added complications in the operating room
		3) Prolonged procedure time
Bronchoscopic transparenchymal nodule access	1) Regardless of the CT bronchus sign, BTPNA can detect PPLs.	1) This technique is invasive.
	2) It has low complications	2) It is more complicated than a transthoracic CT-guided biopsy.
Transbronchial access tool	1) Can help to sample PPLs without bronchus signs.	1) This technique is invasive.
	2) There is a lower risk of adverse events.	2) It is more complicated than a transthoracic CT-guided biopsy.

VBN, virtual navigation bronchoscopy; C-TBNA, conventional transbronchial needle aspiration; ENB, electromagnetic navigation bronchoscopy; BTPNA, bronchoscopic transparenchymal nodule access; PPLs, peripheral pulmonary lesions.

## Author contributions

MG, HD, SG, and JL: conceptualization. MG, HD, SG, and AP: investigation and validation. MG, HD, and AP: project administration. SG and MG: supervision. MG, HD, AP, and JL: writing. MG, HD, SG, AP, and JL: writing—review and editing. All authors contributed to the article and approved the submitted version.

## Funding

This study was supported by Chongqing pulmonary nodule management center (Project Number: 02972018_8_002) and the 2020 Municipal subsidy for training Senior Medical Talents [Project Number: 0202czzx2027 (YC006)].

## Conflict of interest

The authors declare that the research was conducted in the absence of any commercial or financial relationships that could be construed as a potential conflict of interest.

## Publisher's note

All claims expressed in this article are solely those of the authors and do not necessarily represent those of their affiliated organizations, or those of the publisher, the editors and the reviewers. Any product that may be evaluated in this article, or claim that may be made by its manufacturer, is not guaranteed or endorsed by the publisher.
